# Pregnancy-Related Eccrine Angiomatous Hamartoma: Case Report

**DOI:** 10.7759/cureus.52059

**Published:** 2024-01-10

**Authors:** Jonathan David Serrano Arias, Juan Jacobo Del Valle Saavedra, Luz Ellis Arrieta Blanquicet, Ana C Ruiz Suarez

**Affiliations:** 1 Dermatopathology, Universidad CES, Medellín, COL; 2 Dermatology, Universidad CES, Medellín, COL; 3 Dermatology, Promotora Clínica Zona Franca de Urabá, Apatadó, COL; 4 Dermatopathology, Clinica CES, Medellín, COL

**Keywords:** skin diseases, benign vascular tumors, clinico-pathological, eccrine sweat glands, sweat gland neoplasms

## Abstract

Eccrine angiomatous hamartoma is rare, slow-growing, and benign neoplasm that is diagnosed based on clinical characteristics and histological findings. It usually presents as a solitary nodule on the extremities and may arise at birth or in childhood. Although it is usually asymptomatic, in some cases it can cause pain and hyperhidrosis. From a histological perspective, it is characterized by an increase in the number of eccrine glands and a proliferation of vascular channels. We present the case of a 26-year-old woman who developed an eccrine angiomatous hamartoma in her right leg. The rapid growth of the lesion during pregnancy coupled with the challenges posed by a superficial biopsy, complicated the differential diagnosis.

## Introduction

Eccrine angiomatous hamartoma (EAH), also known as eccrine angiomatous nevus, is a rare, benign skin malformation that typically manifests at birth or during childhood, although cases have been documented in adults, with a mean age of 16.4 and 21.1 years [1,2]. Due to its low frequency, there isn't extensive epidemiological information available. It often appears as a solitary pink nodule or plaque on the extremities and is generally asymptomatic, but it may cause pain and hyperhidrosis [3]. The increase in size of EAH is proportional to the patient. Histologically, it is characterized by an increase in the number of eccrine sweat glands and the presence of a significant amount of capillaries in the deep and mid dermis [1,2]. There is an association with other vascular anomalies such as arteriovenous malformations, verrucous hemangioma, and spindle cell hemangioma [2,4]. As it is a benign condition, it generally does not require intervention unless it causes discomfort to the patient in terms of pain, hyperhidrosis, or aesthetic concerns [3,5].

## Case presentation

**Figure 1:**
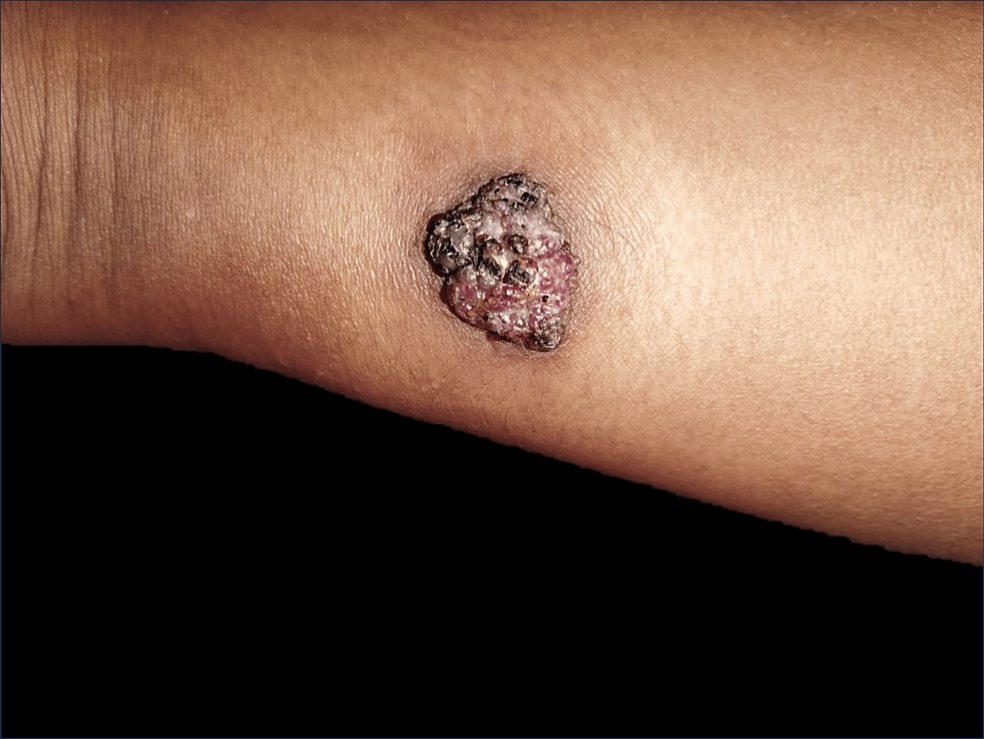
Exophytic tumor, well-circumscribed, black and pink in color, infiltrating the distal and posterior region of the right leg.

**Figure 2:**
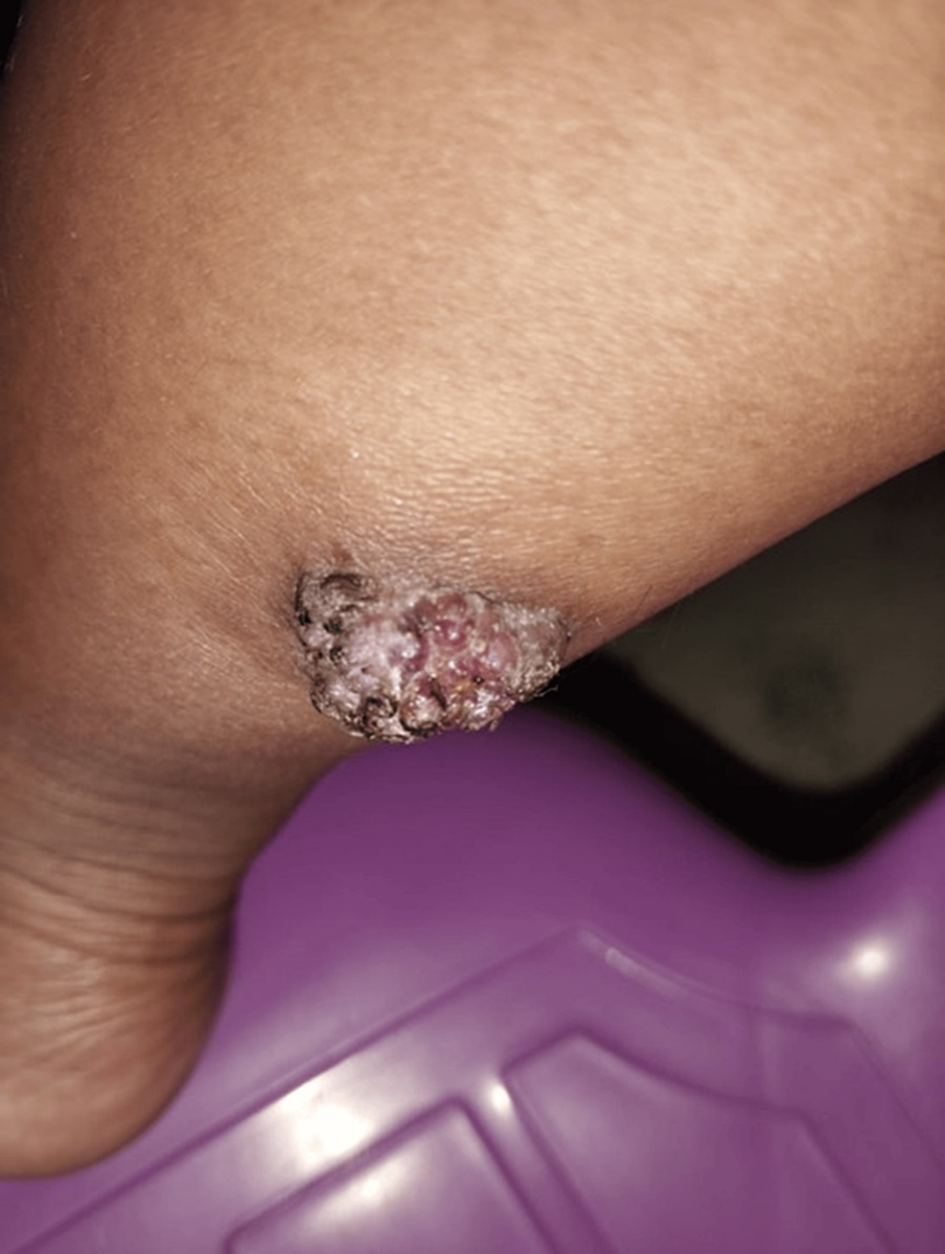
Lateral view of the lesion showing its wart-like appearance with a multilobulated and keratotic surface.

A 26-year-old woman with no significant medical history sought medical attention for an erythematous lesion on the distal and posterior region of her right leg, which had been present since birth. During her first pregnancy, the lesion experienced rapid growth and was accompanied by localized pain and hyperhidrosis. Evaluation revealed a single exophytic tumor, black and pink in color, measuring 30x25 mm in size, well-defined, with keratotic areas, and a multilobulated granulomatous appearance (Figures 1, 2). 

No other abnormalities were identified in the patient's general and systemic examination. Initially, infiltrative lesions were considered as differential diagnoses as sarcoma or squamous cell carcinoma

**Figure 3:**
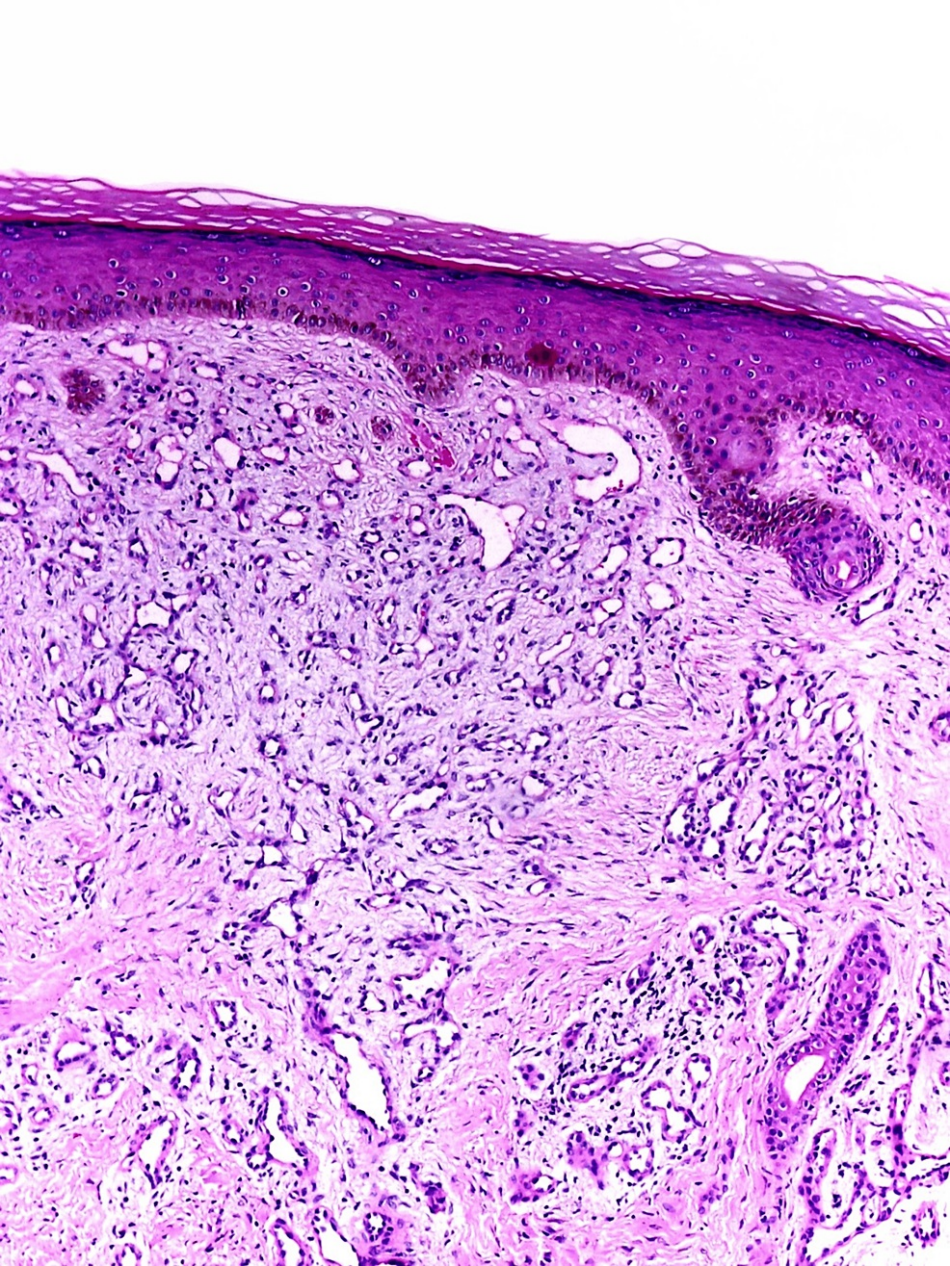
Proliferation of small caliber ectatic vascular structures in the superficial dermis (hematoxylin-eosin stain, original magnification x10).

**Figure 4:**
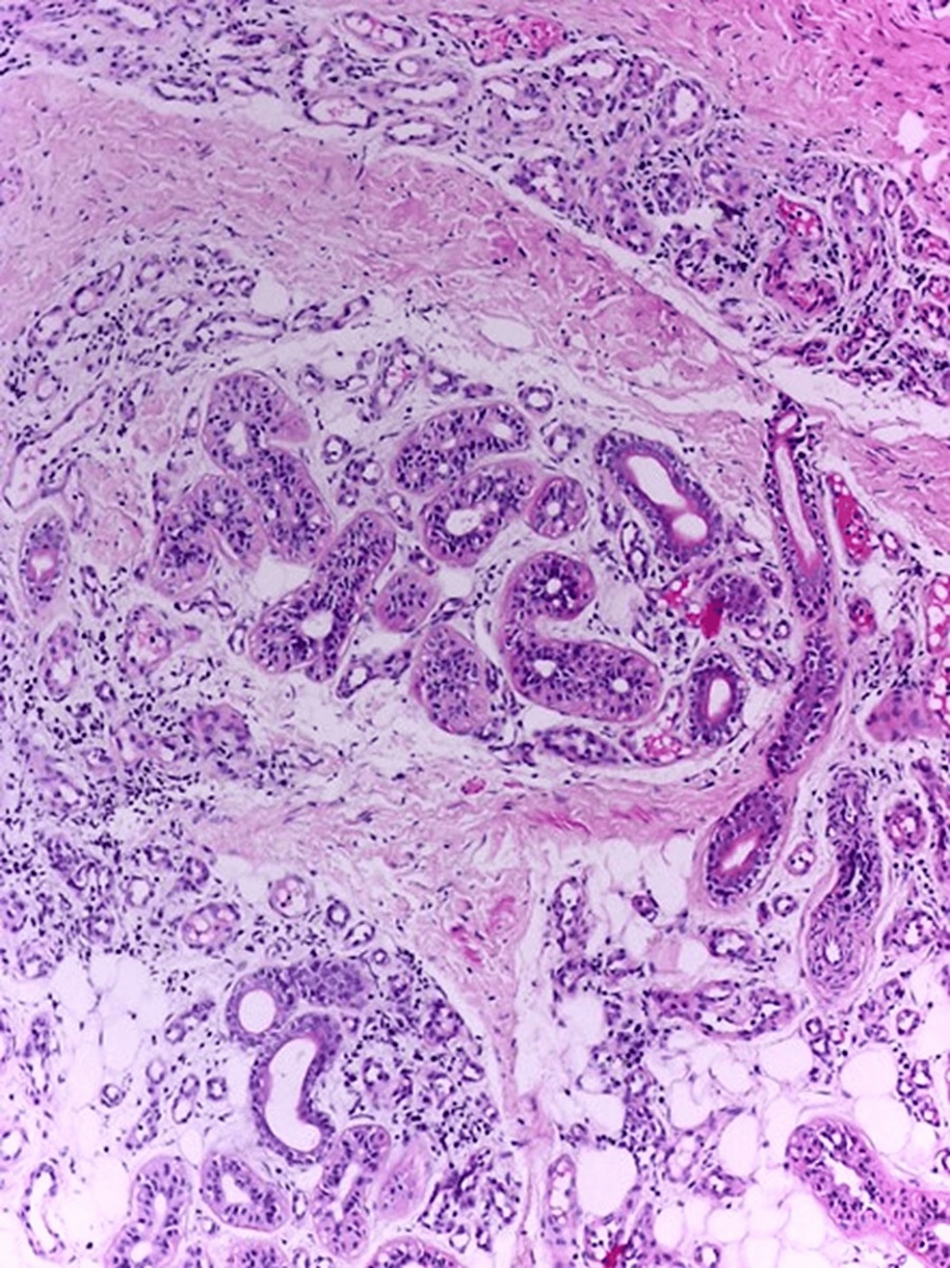
Eccrine angiomatous hamartoma showing small caliber ectatic blood vessels intermixed with multiple eccrine glands and mature adipocytes (hematoxylin-eosin stain, original magnification x10).

A first shave biopsy was performed, resulting in a significant reduction in the size of the lesion, and the hyperhidrosis was limited to the central part of it. The report from this initial biopsy suggested the presence of a pyogenic granuloma. Given the differential diagnoses, a decision was made to conduct another biopsy, this time at a greater depth. The histopathological study of the second biopsy (validated by two pathologists) revealed a lesion that was relatively circumscribed but not encapsulated, composed of a hamartomatous proliferation of secretory eccrine glands and ducts intimately intermixed with a proliferation of small, ectatic blood vessels, the size of a capillary. Additionally, a component of mature adipose tissue was found. The epidermis showed no significant changes, and there was no evidence of atypia or an increase in mitotic activity. With these histological findings, the final diagnosis of EAH was confirmed (Figures 3, 4).

After the second biopsy, the lesion completely regressed and after one year there have been no recurrences.

## Discussion

Eccrine angiomatous hamartoma is characterized by the presence of solitary nodules or plaques, with macular forms in the minority of cases, sometimes with a velvety appearance or verrucous surface, ranging from an average diameter of 3 to 11 cm. Its coloration resembles the patient's skin, although it may present yellowish, pink, blue, or even violet discoloration. There is a predilection for the distal areas of the limbs and palmoplantar regions, although it can be found in other anatomical areas such as the face, neck, buttocks, and sacral region [2,4-6].

In the two most comprehensive retrospective case series in recent years by Sanusi et al. and Patterson et al., which included 26 and 18 cases, respectively, it was observed that EAH occurred from congenital cases up to 84 years, showing a slight male predominance and primarily located on the limbs [7,8]. While several of these characteristics were present in our patient, including the described solitary lesion, the fact that she had a biphasic growth behavior after her pregnancy, and considering that her initial biopsy showed characteristics of a pyogenic granuloma, posed a diagnostic challenge. Additionally, the mentioned case series did not comment on the existence of any biphasic form congenitally, with sudden growth in adulthood related to pregnancy, as was the case with our patient.

Considering that this tumor involves the proliferation of vascular channels, the angiogenic and hormonal factors observed in pregnancy could be a contributing factor to the development of these lesions. Just as in the case reported by Gabrielsen et al., where a 34-year-old woman in her second trimester of pregnancy was diagnosed, following a histopathological study, with EAH on the distal phalanx of the fifth finger of her left hand [9]. As studied in other vascular proliferations, there is an increase in the levels of vascular endothelial growth factor (VEGF) and basic fibroblast growth factor (bFGF), and less circulating tumor necrosis factor-alpha (TNF-α) compared to the control population, which is associated with their formation [10].

The clinical suspicion of a vascular lesion can be confirmed through imaging studies such as nuclear magnetic resonance, ultrasound or color Doppler, but the precise diagnosis requires histology [3,11]. The distinctive microscopic characteristics of EAH include an increase in the number of eccrine glands in the mid and lower dermis, with ectatic or collapsed vessels in relation to hyperplastic eccrine units. This is associated with varying proportions of mature adipose tissue, lymphatics, smooth muscle, pilosebaceous structures, neural elements, and, rarely, bone tissue. The overlying epidermis may be normal or show acanthosis or papillomatosis. Immunohistochemical studies have shown positivity of the eccrine component for S100 and of the vascular component for markers CD31, CD34, and factor VIII [1,2,4,7,12].

In the differential diagnosis, various entities should be considered, such as vascular tumors and vascular malformations, and the approach to painful tumors should not be overlooked [3,13]. In our case, a superficial biopsy was interpreted as a vascular lesion of capillary lobular hemangioma type (pyogenic granuloma), possibly due to the difficulty in appreciating the eccrine glandular component present in the mid and lower dermis. Additionally, eccrine angiomatous hamartoma is a rare lesion and therefore not widely recognized by pathologists and dermatologists. We emphasize the importance of performing a punch biopsy rather than shave biopsies when EAH is suspected. The clinical course of this condition is always benign, with no reports of malignant potential. Regarding treatment, it has been observed that simple excision is often curative, and aggressive resections are not justified [14]. Recently, successful treatment has been achieved using intralesional botulinum toxin and sclerosants [3].

## Conclusions

EAH is a rare condition that should be considered in the management of painful and rapidly growing tumors temporally related to pregnancy. It still has a vascular component that is sensitive to hormones and growth factors related to pregnancy. Despite its benign etiology, due to its rapid growth, it is essential to rule out any of the multiple malignant tumors of the skin adnexa, even in tissues found at greater depths. Deep biopsies are necessary to distinguish between glandular and vascular components since a superficial biopsy may not characterize all the components described in its histopathology. This approach ensures a more precise diagnosis for pregnant patients who require it.
